# *Aggregicoccus* is a myxobacterial genus inherently deficient in fruiting genes

**DOI:** 10.1128/spectrum.02535-25

**Published:** 2026-09-03

**Authors:** Xinran Du, Jianing Wang, Jingjing Wang, Yihan Xu, Qi Chen, Zhiwei Su, Yue-zhong Li

**Affiliations:** 1 State Key Laboratory of Microbial Technology, Institute of Microbial Technology, Shandong University, Qingdao, People's Republic of China; 2 Haide College, Ocean University of China 12591, Qingdao, People's Republic of China; 3 College of Agriculture, Guangxi University 12664, Nanning, People's Republic of China; Indian Institute of Technology Hyderabad, Hyderabad, Telangana, India

**Keywords:** myxobacteria, *Aggregicoccus*, polyphasic taxonomy, fruiting body, comparative genomics

## Abstract

Myxobacteria are fascinating and important prokaryotes with remarkable multicellular behaviors, which make them a model system for studying prokaryotic development and cooperation. Although there have been sporadic discoveries of myxobacterial species unable to fruit, it is unclear whether the non-fruiting characteristic is due to taxon-specific genetic deficiency or suboptimal cultivation conditions. *Aggregicoccus* is a non-fruiting myxobacterial genus typified by a single validly published species, *Ag. edonensis*. In this study, we report five novel *Aggregicoccus* strains, which are classified into three novel type species*, Ag. lacus*, *Ag. agri*, and *Ag. guangxiensis*, based on polyphasic taxonomic analysis. All the *Aggregicoccus* strains are unable to produce fruiting bodies, but can still sporulate. We compared the genome differences between *Aggregicoccus* and *Myxococcus*; both genera belong to the Myxococcaceae family, and all the genomes are of similar sizes. The results showed that the *Aggregicoccus* strains are inherently deficient in the fruiting body-associated genomic information (FAGI). We propose an assessment of FAGI for the classification of non-fruiting myxobacterial species.

**IMPORTANCE:**

Fruiting body formation is traditionally regarded as a defining trait of myxobacteria. Here, we report that *Aggregicoccus* spp., including six strains of four species, can sporulate but are deficient in the fruiting body-associated genomic information (FAGI). This demonstrates that the non-fruiting characteristic in *Aggregicoccus* stems from inherent genetic deficiencies rather than suboptimal cultivation. Our findings highlight the need to assess FAGI presence in classifying non-fruiting lineages, innovate the isolation method, and refine our understanding of the diversity and evolution of the myxobacteria.

## INTRODUCTION

Myxobacteria are a group of gram-negative bacteria that are characterized by multicellular social behaviors (1). The myxobacterial cells are able to migrate in swarms and prey on other microbial cells in a wolf-like pattern (2). Upon starvation, numerous vegetative cells aggregate to develop into species-specific fruiting body structures, wherein the majority of cells lyse, and a minority of cells accounting for approximately 10% of the total ~100,000 aggregated cells in *Myxococcus xanthus* convert to environmentally resistant myxospores (2, 3). Generally, due to the cell density-dependent growth characteristic on complex nutrients or microbial cells (4), myxobacteria cannot be obtained by classical bacterial isolation techniques, such as the dilution spread or streak plate methods; the isolation of myxobacteria generally relies on the formation of fruiting body structures or spreading delicate swarm colonies on plates where soil samples are directly spread over living microbial prey cells or filter papers (5). Cultured myxobacteria are, thus, often able to form fruiting bodies, which are, therefore, considered one of the most important morphological indicators for taxonomic classification of myxobacteria, such as *Chondromyces* for the genus with delicately branched fruiting body structures and *Myxococcus* for the genus with soft spherical types of fruiting bodies (6). However, many newly-identified myxobacterial species are found not to fruit (under standard laboratory conditions), such as *Anaeromyxobacter dehalogenans* (7), *Simulacricoccus ruber* (8), *Aggregicoccus edonensis* (9), *Vulgatibacter incomptus*, and *Labilithrix luteola* (10). It is unclear whether these myxobacteria are truly unable to fruit due to species-specific genetic deficiency or lack of suitable fruiting conditions. More importantly, whether or not fruiting is an overall characteristic for myxobacteria is unclear.

Myxobacterial fruiting bodies are formed on solid surfaces by a high density of cells in response to starvation (1, 11–13). Cell aggregation, fruiting body morphogenesis, and sporulation are three fundamental processes, which are executed by a defined subset of genes (3, 14). Demonstrated in the model myxobacterium *M. xanthus*, cell aggregation during the early fruiting stage is driven by FruA−MrpC-regulated genes (Fmg) and MXAN_2872, which play a key role in coordinating multicellular transition to aggregates (15). Subsequently, fruiting body structures are formed by the aggregated cells in coordination with the help of exopolysaccharide (EPS) and the developmental timers regulating the temporal progression of morphogenesis, which ensures the formation of fruiting bodies in a controlled and ordered manner (16, 17). Finally, spore differentiation within a fruiting body structure is coordinated by the developmental timers realized by the expression of sporulation-associated genes, including the Exo and Nfs sets (18, 19), which is involved in the formation of the spore polysaccharide coat, as well as the *mcu* operon genes and the *dev* operon (15). Although the *dev* operon is not always present in myxobacterial genomes, it contributes to the late-stage regulation of development and sporulation (20). We define these fruiting-associated genes as fruiting body-associated genomic information (FAGI)—“a minimal set of genes directly required for the execution of fruiting body formation.” FAGI emphasizes the genes whose products are directly involved in the three core stages: cell aggregation, fruiting body morphogenesis, and sporulation. Based on this criterion, although regulatory genes, such as *fruA* and *mrpC*, are important, they are not included. Similarly, *csgA* was not included because its product mainly functions in signaling and coordination rather than direct morphogenesis or sporulation (21, 22).

*Aggregicoccus* is a myxobacterial genus established with strains MCy1366^T^ and MCy10622, both belonging to the single species *Ag. edonensis*. Strain MCy1366^T^ was isolated from a Japanese soil sample, and MCy10622 was isolated from a soil sample collected in Switzerland (9). These two strains were described to form cell aggregates or irregularly shaped cell mounds, with no typical fruiting body structures. In this study, we report five novel *Aggregicoccus* strains, which are classified into three novel-type species of the *Aggregicoccus* genus based on analysis of polyphasic taxonomy. We show that *Aggregicoccus* strains are all unable to fruit but can sporulate (9). We compared the genomes of *Aggregicoccus* and the classical fruiting *Myxococcus*, both belonging to the Myxococcaceae family and having similar genome sizes, and revealed that the *Aggregicoccus* strains inherently lack certain essential FAGI genes, leading to an inability to form fruiting bodies. The presence of FAGI was checked in the genomes of other cultured non-fruiting myxobacteria and obtained a similar conclusion (i.e., they are inherently deficient in the development of fruiting body structures). These findings suggest that the inability of *Aggregicoccus* to fruit is a genetic deficiency, and that such a genetic deficiency may represent a common feature among non-fruiting myxobacteria, particularly those uncultured. This prompts a re-evaluation of the universality and utility of fruiting body formation as a taxonomic and screening trait in myxobacteria.

## RESULTS

### Polyphasic taxonomy reveals three novel species of *Aggregicoccus* from five isolates

**Fig 1:**
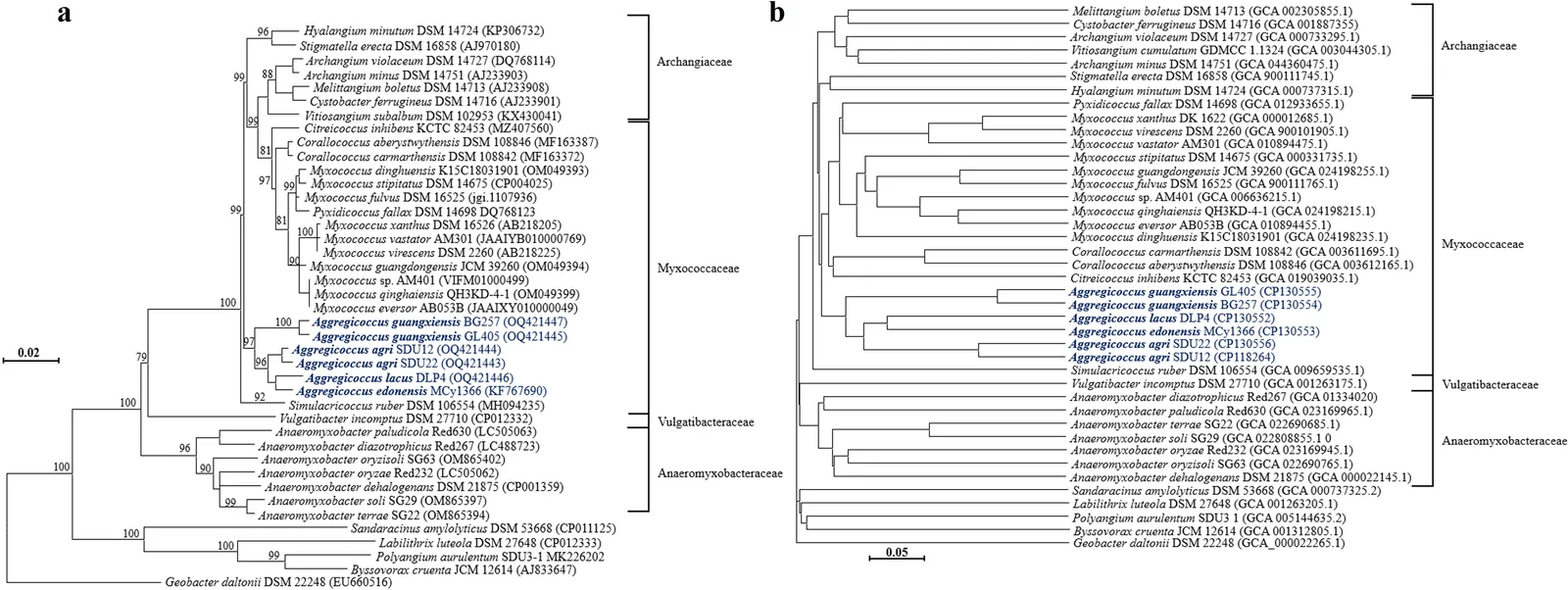
Phylogenetic trees of five novel strains and other closely related species. (**a**) Neighbor-joining phylogenetic tree based on 16S rRNA gene sequences. The scale bar indicates 0.02 substitutions per nucleotide position. The tree is rooted with *Geobacter daltonii* DSM 22248ᵀ as the outgroup. Bootstrap values greater than 75% based on 1,000 replicates are shown at the nodes. (**b**) Whole genome-based phylogenomic tree constructed using CVTree3 with protein sequences and K = 6.

The five myxobacterial strains were isolated from different samples by their myxobacteria-like swarming colonies. These samples were collected from the central campus of Shandong University in Jinan of Shandong Province (strains SDU12^T^ and SDU22), the De-long-pao Lake sediments in Heilongjiang Province (DLP4^T^), and lichen layer of karst landform and seagrass bed sediment in Guangxi Province, China (GL405^T^ and BG257). The 16S rRNA gene sequences of these strains are in a range of 97.28–98.63% identity of the type strain *Ag. edonensis* MCy1366^T^ based on the analysis against the EzTaxon database (Table S1). The neighbor-joining phylogenetic tree based on the 16S rRNA gene sequences revealed that the five myxobacterial strains are in the same phylogenetic cluster with the MCy1366^T^ strain; the cluster can be further divided into three paraphyletic branches: one includes DLP4^T^ and MCy1366^T^; the second contains SDU22 and SDU12^T^; and the third consists of GL405^T^ and BG257 (Fig. 1a). This phylogenetic topology is also supported by the phylogenetic trees reconstructed using the maximum likelihood and maximum parsimony algorithms (Fig. S1), suggesting stable phylogenetic relationships between these *Aggregicoccus* strains.

We sequenced the genomes of six *Aggregicoccus* strains, including the type strain MCy1366^T^ of *Ag. edonensis*, which had not yet been genome-sequenced before this study. Detailed general features of the genome sequences are summarized in Table S2. In agreement with the 16S rRNA gene phylogeny, the *Aggregicoccus* strains are clustered together in the phylogenomic tree; this cluster is clearly separated from the *Myxococcus* genus, as well as other clusters of the Myxococcaceae family (Fig. 1b). Similarly, the *Aggregicoccus* cluster is divided into three distinct branches in the phylogenomic tree. Notably, of the three branches, the 16S rRNA gene sequence identity is 99.41% between SDU22 and SDU12^T^, 99.26% between GL405^T^ and BG257, but 97.68% between DLP4^T^ and MCy1366^T^. According to the threshold of 98.65% for species delineation (23), the five newly isolated myxobacterial strains may represent three novel species of the *Aggregicoccus* genus.

To determine their taxonomic status, we calculated the average nucleotide identity (ANI) and digital DNA–DNA hybridization (dDDH) values pairwise of the six *Aggregicoccus* strains. The ANI and dDDH values were 96.60 and 70.50% between SDU12^T^ and SDU22 and 97.08% and 74.00% between BG257 and GL405^T^, respectively, suggesting that each of the two strain pairs belongs to the same species based on the species delineation thresholds of 95% for ANI and 70% for dDDH (23–25). Comparatively, the ANI and dDDH values were 89.53 and 37.60% for the DLP4^T^ and MCy1366^T^ pair, respectively, both of which were below the thresholds for species delineation. The ANI and dDDH values of the other pairwise comparisons were all below the thresholds (Table S3). Consequently, we propose that the six strains represent four species, and strains DLP4^T^, SDU12^T^ (SDU22), and GL405^T^ (BG257) are phylogenetically affiliated with three novel species of the *Aggregicoccus* genus.

**Fig 2:**
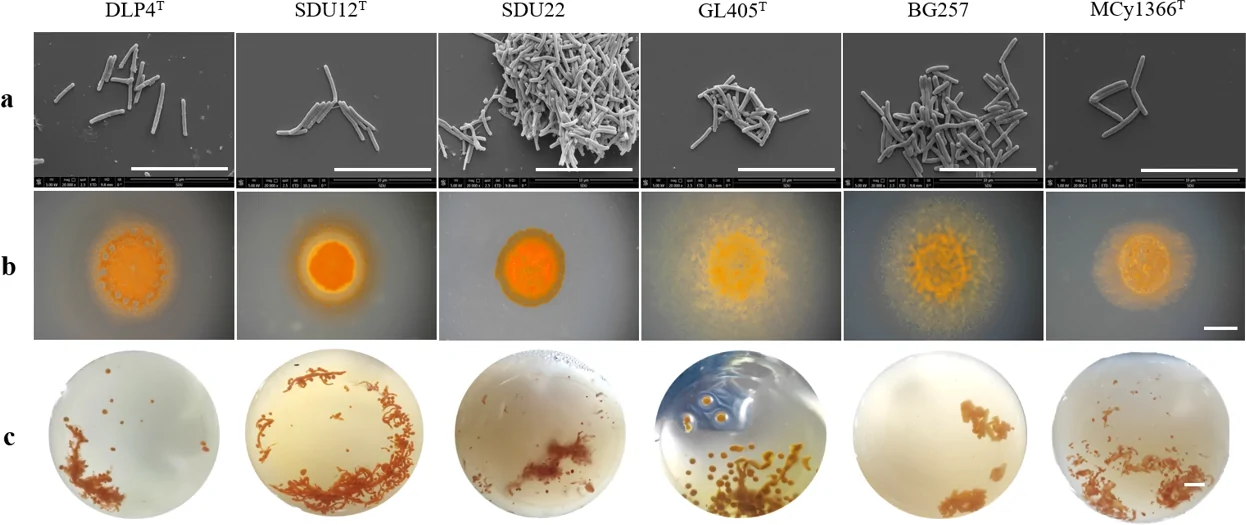
Morphological characterization of the *Aggregicoccus* strains. (**a**) Vegetative cells from the 4-day-old cultures in CY/H broth. Scale bars: 10 μm. (**b**) Colonies on CY/H medium for 4 days. (**c**) Cell clumps in liquid CY/H media for 4 days. The scale bar is 5 mm for panel b and 1 cm for panel c.

These *Aggregicoccus* strains stained gram-negative and Congo red-positive and were positive for catalase reaction (Fig. S2 and S3). They were mesophilic, grew under neutral to weakly alkaline culture conditions (pH 6.0–9.0), and were salt-intolerant (no more than 1.0% NaCl). The vegetative cells of these strains were all long, cylindrical rods with slightly tapered ends that were 4.0–7.0 μm in length and 0.4–0.6 μm in width (Fig. 2a). The swarms of these *Aggregicoccus* strains formed orange, wrinkled, expanding membranes on CY/H agar plates. (Fig. 2b). The cells grew in clumps in liquid CY/H media (Fig. 2c). *Aggregicoccus* strains in liquid shaking cultures could leave flask-attached cell rings at the air interface.

Based on analyses with standard methods for bacterial identification (refer to the Materials and Methods section), the six tested *Aggregicoccus* strains were able to hydrolyze starch, gellan gum, and casein but were unable to hydrolyze chitin, agar, and cellulose. They were incapable of metabolizing glucose, arabinose, mannose, mannitol, N-Acetyl-D-glucosamine, maltose, potassium gluconate, capric acid, adipic acid, malic acid, sodium citrate, and phenylacetic acid for growth. Detected with API reagent strips, all the strains were positive for gelatin liquefaction, esterase (C4), esterase (C8), naphthol-AS-BI-phosphohydrolase, leucine arylamidase, valine arylamidase, acid phosphatase, and arginine dihydrolase, but negative for trypsin, citrate, hydrogen sulfide, urease, and the production of indole. There are also some different physiological and biochemical characteristics between these *Aggregicoccus* strains. For example, the *Aggregicoccus* strains, except for MCy1366^T^, possessed the activity of lipase (C14). DLP4^T^, SDU12,^T^ and SDU22, but not the other three, showed nitrate reduction activity. GL405^T^ and BG257 were inactive, while the other four were active in hydrolyzing chymotrypsin. SDU12^T^ and SDU22 were positive for arginine hydrolase and negative for esculin ferric citrate. More information is shown in Table S4.

Fatty acid composition is an important indicator for bacterial taxonomic classification. The fatty acid compositions of the six *Aggregicoccus* strains cultured in the same growth medium for 48 h are characterized by high concentrations of branched-chain fatty acids but with some differences (Table S5). For example, the major fatty acids (>5% of the total amounts) included iso-C_15:0_ in all the six strains, iso-C_17:0_ in most strains, except for MCy1366^T^, and iso-C_16:0_ only in DLP4^T^. The major fatty acid types of GL405^T^ and BG257 were highly similar to that of MCy1366^T^, except with high contents of iso-C_17:0_. Compared to MCy1366^T^, SDU12^T^ did not include the straight-chain fatty acid C_18:0_ and instead had the branched-chain fatty acid iso-C_17:0_ as a major fatty acid. For DLP4^T^, the major fatty acids were all branched fatty acids, including iso-C_15:0_ (35.8%), iso-C_16:0_ (10.5%), iso-C_17:0_ (6.8%), C_16:1_ω5c (7.4%), and iso-C_17:1_ I /anteiso B C_17:1_ (20.4%).

We also assayed the antibiotic resistance profiles of the six *Aggregicoccus* strains. These strains were all sensitive to vancomycin, ampicillin, cefalothin sodium, oxytetracycline, chloramphenicol, and resistant to rifampin, carbenicillin, and daptomycin. Differently, strains SDU12^T^, SDU22, and MCy1366^T^ were resistant to kanamycin and gentamicin, while the others were sensitive. Strain SDU12^T^ and MCy1366^T^ were resistant to tobramycin, while only strain SDU22 was resistant to spectinomycin; refer to Table S6.

### *Aggregicoccus* strains are non-fruiting myxobacteria with the sporulation capacity

The fruiting body development of myxobacteria is a programmed process with complex regulation (26). Although the advantages are unclear, sporulation within a fruiting body is proposed to be beneficial for population dispersal, germination with massive spore numbers to ensure cell density-dependent growth, and protection of population entities from environmental insults (27). *Aggregicoccus* is established from the single species *Ag. edonensis* using two strains (i.e., MCy1366^T^ and MCy10622 by Sood et al.) (9). The authors tested several media to induce the formation of fruiting bodies but only observed cell aggregates or irregularly shaped cell mounds.

**Fig 3:**
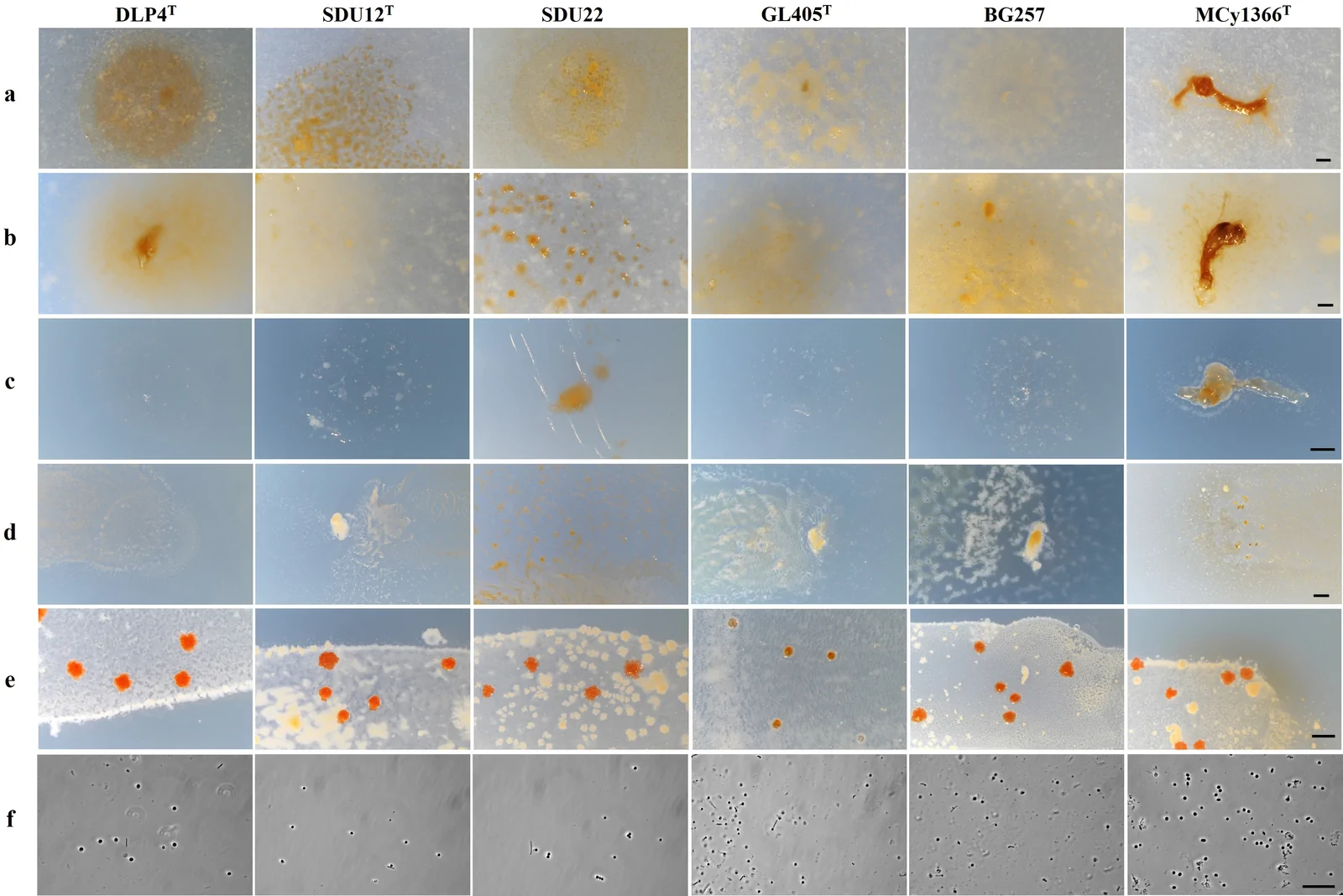
Wild *Aggregicoccus* strains are defective in fruiting body formation—aggregates on plates for 28 days. Scale bars: 1 mm. (**a**) CY agar. (**b**) VY/2 plates. (**c**) TPM plates. (**d**) Water agar-dead *E. coli* plates. (**e**) Water agar-live *E. coli* plates. (**f**) Spherical cells observed under an oil immersion lens after 3 days of induction in 0.5 M glycerol broth. Scale bars: 20 μm. The pictures in **a **to** e **were taken under a stereomicroscope (SMZ143, Nikon). The** f **pictures were taken by a phase-contrast microscope (C-DSD230, Nikon).

Incubating vegetative cells under starvation conditions is a common method used to induce the formation of fruiting bodies in myxobacteria. TPM is a recommended starvation buffer for fruiting body development assay (28). In addition, fruiting bodies can also form after the vegetative growth stage when myxobacterial strains are cultivated on mineral media baited with dead yeast (VY/2) or with living or dead *E. coli* cells (29). In this study, we tested the fruiting potentials of all six *Aggregicoccus* strains under various culture conditions, such as TPM agar, VY/2 agar, water-agar spread with dead or living *E. coli* cells, or CY that was used for cultivating *Ag. edonensis* (9), but all failed to observe the formation of fruiting bodies, even over 8 weeks under 30°C (refer to Fig. 3a through e).

Myxobacterial vegetative cells can undergo chemically induced sporulation in response to chemicals, such as glycerol, propanol, ethylene glycol, phenethyl alcohol, and dimethyl sulfoxide, without the formation of fruiting bodies (1, 30, 31). For the non-fruiting *Ag. edonensis* strains MCy1366^T^ and MCy10622, Sood et al. also found that spherical cells could be induced by shaking the vegetative cells in a myxo media broth (9). We confirmed that shaking each of the six *Aggregicoccus* strains in 0.5 M glycerol broth (1% Casitone, 8 mM MgSO_4_, and 0.5 M glycerol) (30) or the myxo media broth induced the conversion of cells from long vegetative type to spherical and refractive spore type. Notably, these spherical cells often coexisted with many rod-shaped vegetative cells in different degrees for different *Aggregicoccus* strains (Fig. 3f exemplifies the results induced with glycerol). The spherical cell resuspensions were heat-treated at 50°C for 2 h, which can kill the vegetative cells (29). The treated cells were then spread with 0.3–0.4% soft agar on VY/2 plates and incubated at 30°C. We found that all the tested strains regrew, proving thermoresistant capacities of the spherical cells as spores. We concluded that all these *Aggregicoccus* strains can sporulate without the formation of fruiting bodies under some incubation conditions, such as glycerol broth or myxo media broth.

### *Aggregicoccus* strains are genetically deficient in the formation of fruiting bodies

The genetic mechanisms known to date for fruiting body formation are mainly from *Myxococcus xanthus*, the most extensively studied model species of myxobacteria (1, 26, 32). The non-fruiting *Aggregicoccus* is a paraphyletic genus of *Myxococcus* in the Myxococcaceae family. To investigate the potential genetic mechanisms underlying the fruiting body formation deficiency in *Aggregicoccus*, we compared the genomes of the six *Aggregicoccus* strains (Table S2) with genomes of the type strains of 10 *Myxococcus* species, including *M. xanthus* (Table S7). All these *Myxococcus* species and strains have been reported to have the capacity of forming fruiting bodies containing myxospores (6, 33, 34). The genomes of *Aggregicoccus* and *Myxococcus* strains are both in a large genome size (8.23–9.69 Mbp vs*.* 8.99–12.41 Mbp) and have a high guanine–cytosine (GC) content (69.99–70.94% vs. 68.50–71.00%). Similar genome sizes suggest that the *Aggregicoccus* strains may have potential to fruit as the *Myxococcus* strains, yet they do not.

**Fig 4:**
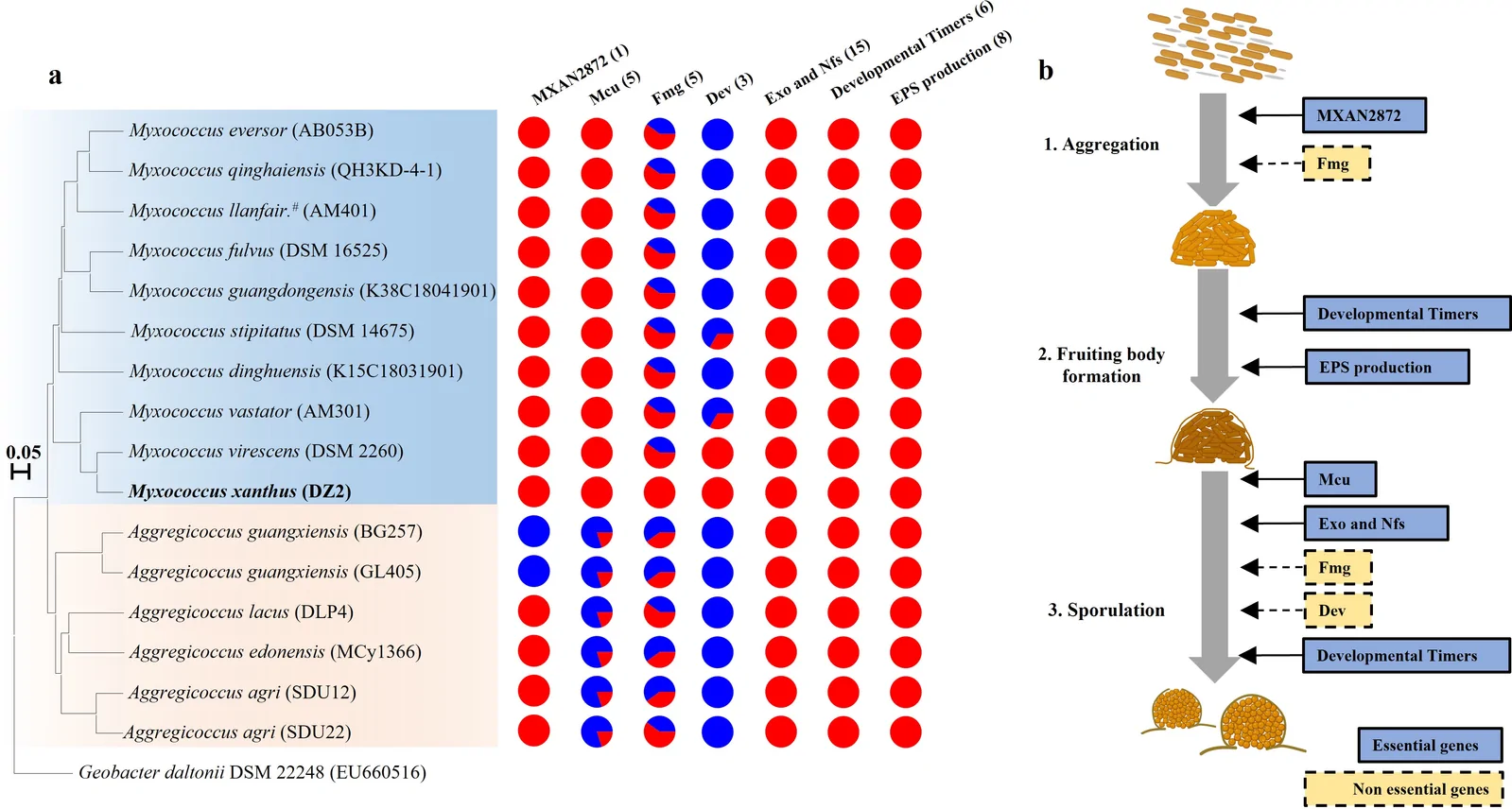
Comparative genomic analysis revealed the mechanisms underlying fruiting body formation. (**a**) The phylogenetic tree on the left was constructed based on whole-genome sequences using CVTree3 (*K* = 6). The numbers in parentheses on the right indicate the number of genes in each category. Red indicates the presence of these genes in the corresponding genome, while blue indicates their absence. Detailed gene information was provided in Table S6. The species name *Myxococcus llanfairpwllgwyngyllgogerychwyrndrobwllllantysiliogogogochensis* is abbreviated as *Myxococcus Llanfair* and labeled with “#.” (**b**) Essential and non-essential genes for fruiting body formation inferred from comparative genomic analysis.

The development of fruiting body structures involves three essential processes: cell aggregation, fruiting body morphogenesis, and sporulation together with cell lysis. We summarize the fruiting body-associated genomic information (FAGI) distribution in the *Aggregicoccus* and *Myxococcus* genomes (Table S8). We noticed that, without regard for the gene sequence variation, expression regulation, and functional activation, each of the *Myxococcus* genomes possesses complete gene sets of MXAN_2872, Mcu, Exo, and Nfs, EPS production, and developmental timers; some *Myxococcus* species partially lose some genes of the Fmg and Dev sets that are present in *M. xanthus* (Fig. 4a). The systematic loss suggests that the Fmg and Dev sets, at least the lost genes, are probably non-essential for the fruiting body formation in *Myxococcus* species other than *M. xanthus*. Comparatively, the homologous genes encoding for Exo and Nfs, EPS production, and developmental timers were found in all the non-fruiting *Aggregicoccus* strains. Similar to that in most *Myxococcus* species, all the *Aggregicoccus* strains completely lost the Dev gene set and partially lost the Fmg genes. Interestingly, all the *Aggregicoccus* strains partially lost the Mcu genes, and the two strains of *Ag. guangxiensis* completely lost MXAN_2872, while the four strains of the other three *Aggregicoccus* species possessed the gene. The systematic loss, probably species-specific, of essential genes for fruiting body formation is a potential reason for the non-fruiting characteristic of *Aggregicoccus* (Fig. 4b).

Cell motility is obviously the basis for fruiting body formation in myxobacteria. The gliding motility of *Myxococcus xanthus* refers to the smooth movement of individual cells on solid surfaces, namely A-motility. In contrast, S-motility is essentially a type IV pili-dependent group spreading behavior (35). Among the 18 genes associated with A-motility (36–41), all six *Aggregicoccus* strains carried the full set. For the 14 genes related to S-motility (42–49), strains BG257, SDU12^T^, SDU22, and GL405^T^ lacked *masA* and *masB* (detail in Table S8). The *masA* and *masB* genes are core components of the *masABK* operon; they form a membrane-bound complex and interact with PilA—the key subunit of type IV pili (T4P)—thereby regulating S-motility, EPS synthesis, and fruiting body development in *Myxococcus xanthus* (49). Notably, *Aggregicoccus* strains do not exhibit an S-motility-defective phenotype (Fig. S4).

In addition to *Aggregicoccus*, several myxobacterial species have been also reported to be unable to fruit (7, 8, 10, 34, 50–53). Among these culturable non-fruiting myxobacteria, the genomes of some species are large, such as *Polyangium aurulentum* SDU3-1^T^ (12.32 Mbp), *Sandaracinus amylolyticus* DSM 53668^T^ (10.33 Mbp), *L. luteola* DSM 27648^T^ (12.19 Mbp), and *Ag. edonensis* MCy1366^T^ (9.69 Mbp), while others are comparatively small, including the *Anaeromyxobacter* strains (from 3.26 Mbp to 6.72 Mbp), *V. incomptus* DSM 27710^T^ (4.35 Mbp), and *Sim. ruber* 17bor-14^T^ (6.93 Mbp) (Table S9). We also checked FAGI in the genomes of these non-fruiting myxobacteria and revealed that, regardless of whether these had large or small genome sizes, these non-fruiting myxobacteria are always deficient in some essential genes to different extents for fruiting body formation (Table S10). For example, *An. paludicola* Red630^T^ and *An. dehalogenans* 2CP-1^T^ both have the complete Mcu genes but lose many other FAGI genes. Similarly, *V. incomptus* DSM 27710^T^ and *Sim. ruber* 17bor-14^T^ have the complete Exo and Nfs sets, while other non-fruiting strains do not. Notably, these non-fruiting myxobacterial species were generally identified on the basis of one or a few strains. Because there are no sufficient representatives, we could not summarize FAGI of the associated taxa. However, the universal genetic deficiency in the fruiting process of non-fruiting myxobacteria highlights that such a deficiency is probably a common characteristic for many uncultured myxobacteria, although the presently cultured myxobacteria are mostly able to fruit. For example, two metagenome-assembled genomes (6.15 ± 1.28 Mbp and lower gene count 5,129 ± 1,005) of anaerobic non-soil uncultured Myxococcota exhibited severely curtailed social behavior pathways and missing homologues of genes specific for the typical social lifestyle of myxobacteria (21).

**Fig 5:**
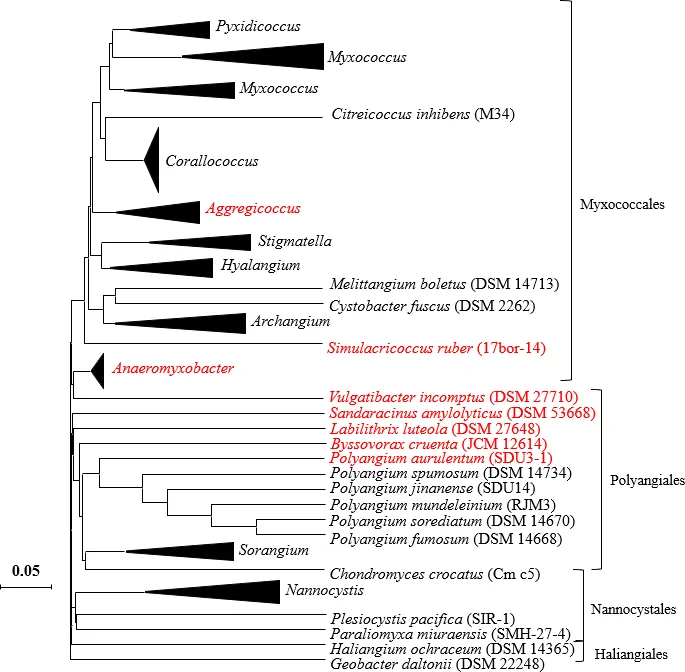
Phylogenomic tree of cultured myxobacteria genera. The tree was constructed using CVTree3 with protein sequences and *K* = 6. The red clusters represent non-fruiting genera that have been reported (species details refer to Fig. S5).

We constructed a genomic tree containing the type strains of all the cultured fruiting and non-fruiting myxobacterial taxa (Fig. 5; details refer to Fig. S5). We found that the non-fruiting myxobacteria with small genome sizes, including *V. incomptus* DSM 27710^T^, *Sim. ruber* 17bor-14^T^, and the *Anaeromyxobacter* spp., all located at the root of the Myxococcales order; the non-fruiting *San. amylolyticus* DSM 53668^T^, *L. luteola* DSM 27648,^T^ and *Byssovorax cruenta* JCM 12614^T^, which have large genome sizes, were all at the root of the Polyangiales order. Similarly, the non-fruiting *Aggregicoccus* spp. were at the root of the family *Myxococcaceae,* while *P. aurulentum* SDU3-1^T^ was at the root of the *Polyangium* genus. The phylogenetic relationships between fruiting and non-fruiting myxobacteria all support that the non-fruiting myxobacteria are ancient stages evolving toward the different fruiting myxobacterial taxa. Further investigations on the non-fruiting characteristics of *Aggregicoccus* strains, as well as other non-fruiting myxobacteria, will reveal the evolution of fruiting trait within this specific social bacterial group.

### Bacteriolytic activities, antifungal activities, and ecological distribution of *Aggregicoccus* strains

Myxobacteria can be divided into bacteriolytic and cellulolytic groups because of their degradation ability on living microbial cells or cellulose (29). We determined that all six *Aggregicoccus* strains are unable to degrade cellulose but exhibit, although with some differences, robust predation abilities on different bacterial cells (Fig. S6). For example, all the *Aggregicoccus* strains were capable of preying on *Staphylococcus aureus* DSM 20231 and *Micrococcus luteus* ATCC 4698, but none of them grew on the *Pseudomonas aeruginosa* PAO1 prey mats. On living *Escherichia coli* DH5α, strains SDU12^T^, SDU22, BG257, and GL405^T^ grew well, while DLP4^T^ and MCy1366^T^ exhibited weak predation ability. BG257 and GL405^T^ exhibited the predation ability on *Acinetobacter baumannii* ATCC 19606, while the other four tested strains had no such activity. In addition to the predation activity on bacterial cells, all the *Aggregicoccus* strains exhibited inhibitory effects on the growth of fungal hyphae (Fig. S7).

Although not clearly elucidated, previous studies indicated that the wolf pack-like predation of *M. xanthus* relies on the collection of extracellular enzymes (4). Consistently, CAZy family genes accounted for about 3.6–3.9% of the *Aggregicoccus* genomes, including plenty of glycosyl hydrolase (GH) families, glycosyl transferases (GT) family genes, and carbohydrate-binding modules (CBM) family genes. Fig. S8 exemplified the compositions of glycosyl hydrolases (GHs), GluM (outer membrane β-1,6-glucanase, which acts on the cell walls of mycelia [54]), and other predation-specific genes (55). Rich predation-specific genes may enable effective resource acquisition and competitive dominance (54, 56). Notably, the recently discovered killing systems for predation (i.e., the contact-dependent “Kil” system [Tad]) (57) and the type 3-like secretion system are also present in these *Aggregicoccus* genomes (refer to Table S11).

Myxobacteria have garnered widespread attention for their extraordinary ability to produce secondary metabolites, which not only serve as a valuable source for drug screening (58, 59) but also provide myxobacteria with a strong capacity to fight against other microbes. Diverse biosynthetic gene clusters (BGCs) are also detected in the six *Aggregicoccus* genomes, including type I and III polyketides (PKs), non-ribosomal peptides (NRPs), terpenes, lanthipeptides, thioamitides, and microviridins (Table S12). The BGCs of the six strains from different habitats showed different characteristics probably associated with their habitat adaptation characteristics. For example, the specific gene clusters of SDU12^T^ and SDU22 from campus garden soil were mainly arylpolyene, resorcinol, and betalactone, while BGCs of acyl_amino_acids and thiopeptide were exclusive to GL405^T^ and BG257, which live in coastal habitats. DLP4^T^, GL405^T^, and BG257 had more thioamitides, LAP, T1PKS, RiPP-like, T3PKS, and microviridin than other strains from soil habitats. DLP4^T^ from lake sediments shares the common BGC characteristics of soil and coastal strains.

Culture-independent surveys show that myxobacteria are widely distributed and among the most predominant bacterial groups in many natural environments, but different myxobacterial taxa usually have different habitat preferences (60–62). Considering the rigid conditions for fruiting body formation, the ubiquity and diversity of myxobacteria suggest that the fruiting is not a common feature among diverse myxobacteria. We further analyzed the distribution and proportion of *Aggregicoccus* in worldwide environmental samples from the Microbe Atlas Project (MAP) data (63). Shown by the appearance of amplicon sequence variants (ASVs) obtained in environmental samples, *Aggregicoccus* clearly prefers soil habitats, being detectable in 46.06% of the 20,654 soil samples, followed by the plant-related samples (21.06% of the total 9,598 samples). *Aggregicoccus* was also detected in aquatic (7.98% of 8,700 samples) and animal-related (2.53% of 75,079 samples) environments (Fig. S9).

### Description of novel *Aggregicoccus* species

The above polyphasic taxonomic analysis with the reference strain *Ag. edonensis* MCy1366^T^ determined that the five novel myxobacterial strains belong to three novel species within the *Aggregicoccus* genus. We propose to establish three novel species affiliated within *Aggregicoccus: Ag. lacus*, with DLP4^T^ as the type strain; *Ag. agri*, with SDU12^T^ as the type strain; and *Ag. guangxiensis*, with GL405^T^ as the type strain.

### *Aggregicoccus lacus* sp. nov

#### *Aggregicoccus lacus* (la′cus. L. gen. masc. n. *lacus*, of a lake)

Vegetative cells are rod shaped and gliding motile on agar medium; Gram stain negative, aerobic, and catalase and oxidase positive. Swarm colonies are orange on CY/H plates or metallic pink on VY/2 agar with no diffusing pigment and positive in Congo red stain. Typical fruiting body does not form. Growth occurs at pH 6.0–10.0 (optimum 7.0–7.5), 18–45°C (optimum 30°C), and with 0–1.5% NaCl (w/v, optimum 0%). It utilizes starch, gelatin, and esculin but not agar, cellulose, and chitin. Nitrate reduction is positive. Resistant to rifampin, carbenicillin, and daptomycin. Major fatty acids contain iso-C_15:0_ (35.8%), iso-C_16:0_ (10.5%), iso-C_17:0_ (6.8%), C_16:1_ω5c (7.4%), and iso-C_17:1_ I /anteiso B C_17:1_ (20.4%).

The type strain is DLP4^T^ (= KCTC 92618^T^ = CGMCC 1.61702^T^) isolated from De-long-pao Lake sediments collected from China. The genome is 9.15 Mb, and the DNA G + C is 70.07 mol%. GenBank accession number for the 16S rRNA gene sequence and genome are OQ421446 and CP130552, respectively.

### *Aggregicoccus agri* sp. nov

#### *Aggregicoccus agri* (a′gri. L. gen. masc. n. *agri*, of a field)

Vegetative cells are gram negative and rod shaped. Cells are gliding motile on agar medium. Swarm colonies are thin, pale yellow to pink-orange on CY/H agar and positive in Congo red stain. Typical fruiting body does not form. Growth occurs at pH 6.5–10.0 (optimum 7.0–7.5), 18–40°C (optimum 30°C), and with 0–1.5% NaCl (w/v, optimum 0%). Catalase and nitrate reduction are positive, while oxidase and urease are negative. It hydrolyzes gelatin, casein, and starch but does not utilize chitin, agar, esculin, or cellulose. Major fatty acids contain C_16:0_ (15.2%), iso-C_15:0_ (16.9%), iso-C_17:0_ (16.9%), C_18:1_ω9c (17.5%), and C_16:1_ω7c /C_16:1_ω6c (18.8%). It is resistant to rifampin, kanamycin, carbenicillin, gentamicin, and daptomycin.

The type strain is SDU12^T^ (= KCTC 92617^T^ = CGMCC 1.61704^T^) isolated from upper soil sample collected from Jinan campus garden of Shandong University in China. The genome is 8.4 Mb, and the DNA G + C is 70.0 mol%. GenBank accession number for the 16S rRNA gene sequence and genome are OQ421444 and CP118264, respectively.

### *Aggregicoccus guangxiensis* sp. nov

#### *Aggregicoccus guangxiensis* (guang.xi.en′sis. N.L. masc./fem. adj. *guangxiensis*, referring to Guangxi)

Vegetative cells are slender rod shaped. Swarm colonies are golden yellow to orange on CY/H medium with lustrous and veined wrinkles and very weakly positive in Congo red stain. Cells are gliding motile on agar medium and Gram stain negative, aerobic, catalase positive, and oxidase negative. Typical fruiting body does not form. Growth occurs at pH 6.0–10.0 (optimum 7.0–7.5), 18–35°C (optimum 30°C), and with 0–1.5% NaCl (w/v, optimum 0%). It is able to hydrolyze gelatin, starch, casein, and esculin, but not chitin, agar, and cellulose. Enzyme activities were positive for esterase (C4), esterase (C8), lipase (C14). Negative for urease, nitrate reduction, trypsin, and indole. Major fatty acids contain C_16:0_ (13.6%), C_18:0_ (5.3%), iso-C_15:0_ (15.8%), iso-C_17:0_ (16.0%), and C_16:1_ω7c /C_16:1_ω6c (23.2%). It is resistant to rifampin, carbenicillin, and daptomycin.

The type strain is GL405^T^ (= KCTC 92742^T^ = CGMCC 1.61703^T^) isolated from lichen layer of karst geological formation rocks in Guilin City, Guangxi Province, China. The genome is 8.23 Mb, and the DNA G + C is 70.9 mol%. GenBank accession number for the 16S rRNA gene sequence and genome are OQ421445 and CP130555, respectively.

## DISCUSSION

Based on cultured and uncultured myxobacteria detected in a campus soil of Shandong University, we found that, together with a few culturable fruiting myxobacterial taxa, many uncultured myxobacteria are present in the single soil sample; we made a perspective that the myxobacteria in nature exist in two forms—fruiting and non-fruiting living together in soil habitats (64). In fact, many non-fruiting myxobacteria have been isolated, and almost all of them are from soil. We do not know the living mechanisms of the myxobacteria without the fruiting ability, which requires deep investigations.

In recent years, many non-fruiting taxonomic groups of myxobacteria have been reported, but little is known about the non-fruiting properties. Analyses on metagenome-assembled genomes of uncultured myxobacteria have not only revealed their unusual characteristics, such as low genomic GC content (21) and photosynthesis (65), but also shown fruiting deficiency. We speculate that deficiency in genetics for the fruiting body formation might be a normal characteristic for many myxobacteria, especially those that remain uncultured. To taxonomically classify novel myxobacteria deficient in fruiting body formation, we propose to perform a genetic deficiency analysis of FAGI, in addition to fruiting assays under various conditions. This study can provide a reference for future investigations on non-fruiting myxobacteria.

The discovery of inherent deficiency in genetics of *Aggregicoccus* species and strains in fruiting body formation offers an insight into the evolutionary dynamics and ecological adaptations of myxobacteria. Fruiting myxobacteria possessing more complicated genomes may represent a evolutionary strategy, which tends to require more growth patterns to cope with complex environments (64, 66). Based on the culture-independent survey, the cultured myxobacterial taxa account for approximately 10% of all the detected myxobacteria at the species level (60). The low degree of cultivation of myxobacteria is probably related to the limitations of the classical isolation methods, which primarily rely on the formation of fruiting bodies, induced directly from environmental samples (2). Innovation in the isolation methods for myxobacteria should be independent from the fruiting characteristic. Again, investigations on the non-fruiting *Aggregicoccus*, as well as other non-fruiting myxobacteria, will reveal some specific characteristics of myxobacteria, which will be the basis for isolation method innovation.

## MATERIALS AND METHODS

### Isolation and culture conditions

All the five strains were screened by using the standard method with living *Escherichia coli* as the prey bait smeared over the water agar plate and isolated based on their myxobacterial type of colonies (i.e., swarming in a thin film). The type strain of *Aggregicoccus edonensis* MCy1366^T^ (DSM 27872) was obtained from the DSMZ (German Collection of Microorganisms and Cell Cultures). All the six strains were cultured on VY/2 agar (5 g/L dried baker’s yeast, 1.0 g/L CaCl_2_·2H_2_O, 15 g/L agar, pH 7.2) or CY/H medium (1.0 g/L defatted soy flour, 1.0 g/L glucose, 4.0 g/L starch, 1.5 g/L yeast extract, 1.5 g/L casitone, 1.0 g/L CaCl_2_·2H_2_O, 0.5 g/L MgSO_4_·7H_2_O, 0.008 g/L iron–EDTA, 11.8 g/L HEPES, pH 7.3, 15 g/L agar) for further characterization. The myxo media broth (10 g casein peptone, 1 mg CoCl_2_, 0.05 g CaCl_2_·2H_2_O, 0.25 g MgSO_4_·7H_2_O, 23.6 g HEPES, 1 L distilled water; pH 7.0) and 0.5 M glycerol broth (1% Casitone, 8 mM MgSO_4_, and 0.5 M glycerol) were shaken to test sporulation.

### Phenotypic characterization

Morphological characteristics of the cultures grown on CY/H medium at 30°C were documented. Colonies were photographed under a stereomicroscope (SMZ143, Nikon). Vegetative cells and myxospores were observed by using a phase-contrast microscope (C-DSD230, Nikon) or a scanning electron microscope (SEM) (Quanta 250 FEG; FEI).

### Genome sequencing and analysis

Genome DNA was extracted using a TIANamp Bacteria DNA Kit (TIANGEN) according to the manufacturer’s protocols. The complete genomes of six strains were sequenced using *de novo* sequencing with DNBSEQ platform and Nanopore platform, which were assembled by BGI Genomics Co., Ltd. (Shenzhen). The average nucleotide identity (ANI) values between isolates and the selected taxa were determined using the online ANI calculator (https://www.ezbiocloud.net/tools/ani). DNA–DNA hybridization (DDH) was calculated by using the Genome–Genome Distance Calculator software (version 2.1; https://ggdc.dsmz.de/distcalc2.php) (25). The genome sequences were searched against the databases of COG (http://www.ncbi.nlm.nih.gov/COG/), Pfam-A (v32.0) (http://pfam.xfam.org/), and CAZy (67) to functionally annotate genes, families, and domains. The amino acid sequences of development-related genes were searched in the National Center for Biotechnology Information (NCBI) gene database.

Although fruiting genes are mainly studied in *M. xanthus* DK1622, it is important to note that DK1622 is not a wild-type strain, but a lab-generated “reconstructed wild type,” which misses some genes in comparison with the real wild-type strain, *M. xanthus* DZ2 (68). We also conducted a comparative genomic analysis on DZ2 (Ga0559841_01 and Ga0560123_01) and confirmed that the developmental genes discussed in this study are conserved in DZ2. In this study, for convenience, gene annotations and locus tags are based on the *M. xanthus* DK1622 genome (MXAN), which is often employed in references.

### Phylogenetic analysis

The 16S rRNA gene sequences were amplified with the universal bacterial primers 27F and 1492R (69). Sequence similarity was searched using NCBI-BLAST Nucleotide Database and the EzBioCloud server (70). Phylogenetic trees of the 16S rRNA gene sequences were constructed using the neighbor-joining, maximum parsimony, and maximum-likelihood methods in the software package MEGA11 (71). The topology robustness of the phylogenetic trees was evaluated with the bootstrap analysis of 1,000 replicates. The whole genome-based phylogenomic tree was constructed based on the genome protein sequences using CVTree3 (72). The reference genome sequences used were all obtained from the NCBI Genome database.

### Physiologic and chemotaxonomic characteristics

The growth ability with different temperatures was performed on VY/2 plates for 3–7 days. The pH range was adjusted to 5.0–10.5 (at an interval of 0.5 pH units) buffered with MES (pH 5.0–6.0), MOPS (pH 6.5–8.0), tricine (pH 8.5), and DHES (pH 9.5–10.5). Tolerance to NaCl (0, 0.5, 1, 1.5, 2, 3%; w/v) was tested similarly on VY/2 agar plates at 30°C for 7 days. The anaerobic growth ability was assayed by incubating the strains on VY/2 medium at 30°C for 2 weeks in an anaerobic incubator.

Gram-staining reaction was tested using a Gram stain kit (Huankai) according to the manufacturer’s instructions. The plate cultures were flooded with an aqueous 0.01% (w/v) Congo red solution for 5–10 min for staining (73). Hydrolysis of gelatin (1.5%, w/v), skimmed milk (2%, v/v), chitin (10% w/v), sodium carboxymethylcellulose (1%, w/v), and Tween 20, 40, 60, and 80 (1%, w/v) were tested on supplemented CY/H plates at 30°C for 14 days (74). For the oxidase reaction, 1% (w/v) aqueous solution of N, N, N′, N′-tetramethyl-p-phenylenediamine dihydrochloride was directly dropped on a colony. A color change to rose red or dark purple means a positive reaction. Catalase activity was detected by the production of bubbles following the addition of a drop of 3% (v/v) H_2_O_2_ (75). To determine the antibiotic resistance, 12 antibiotics were each filter-sterilized and added into pre-cooled VY/2 medium to the final concentration of 50 μg/mL according to references (50, 76), and the medium without antibiotics was used as control. All the above experiments were performed in triplicate.

Other physiological and biochemical characteristics were determined by using the API ZYM, 20E, and 20NE kits (bioMérieux) following the manufacturer’s instructions. Fatty acids were extracted from the cells grown on buffered VY/2 medium at 30°C for 5 days according to the standard protocols of MIDI (Sherlock Microbial Identification System, version 4.5). Fatty acids were analyzed by using an Agilent HP6890 gas chromatograph and identified with the TSBA40 database of the microbial identification system (77). The other physiological and biochemical properties were tested by using the API 20E and 20NE reagent strips based on CY/H medium. The extracellular enzymatic activities were assayed by using the API ZYM reagent strips according to the instructions.

### Comparative genomic analysis

After constructing an index using makeblastdb, we performed blastp (e-value < 10^−10^) to compare these genes against the 10 type strains of *Myxococcus* capable of forming fruiting bodies (2, 14, 33, 76, 78–80), the six *Aggregicoccus* strains, and the 13 non-fruiting myxobacterial strains reported in the literature (7, 8, 10, 34, 50–53).

### Predation and antifungal activity

To investigate microbial predation on living microorganisms, including gram-negative *E. coli* DH5α, *Acinetobacter baumannii* ATCC 19606, and *Pseudomonas aeruginosa* PAO1, gram-positive *Staphylococcus aureus* DSM 20231 and *Micrococcus luteus* ATCC 4698 were tested for 2-week incubation on water agar medium at 30°C. The cells were suspended in physiological saline. The cell suspensions were concentrated 30× for the bacterial prey and 10× for tested myxobacterial cells. A volume of 50 µL of the prey bacterial cell suspension was spotted on water agar medium. Then, a volume of 3 µL of the tested myxobacterial strains was spotted on the center of the prey lawn. The colonies were photographed every 48 h under a stereomicroscope (SMZ143, Nikon).

The freshly cultured strains of *Aggregicoccus* and fungal spores were suspended with 0.85% (w/v) NaCl, and homogenized cell suspension was obtained by grinding the *Aggregicoccus* tissue at low speed using a tissue grinder. The fungal spore suspension was spotted in the center of the TPY plates, and the *Aggregicoccus* cell suspension was spotted around the fungal spore point at a distance of 1 cm, with four spots per plate. TPY plates were prepared with TPM buffer with 0.6 g/L potato extract, 2 g/L glucose, 2 g/L yeast powder, and 15 g/L agar. The control was not inoculated with *Aggregicoccus*, and all plates were incubated in triplicate at 30°C for 1 week to observe the growth of the colonies. The colonies were photographed under a 1× magnification of stereomicroscope (SMZ143, Nikon).

### Analysis of habitat distribution characteristics

To analyze the habitat distribution characteristics of *Aggregicoccus*, its 16S rRNA sequences were used to search the MAP database across four habitats—soil, aquatic, plant, and animal—using an identity threshold of >0.97 (63). Samples with more than 10,000 reads were considered, and the occurrence frequency of *Aggregicoccus* was calculated as the number of samples in which *Aggregicoccus* was detected divided by the total number of samples.

## Data Availability

The GenBank/EMBL/DDBJ accession numbers for the 16S rRNA gene sequences are OQ421446 (DLP4^T^), OQ421444 (SDU12^T^), OQ421443 (SDU22), OQ421445 (GL405^T^), and OQ421447 (BG257). The genome sequence data are deposited in DDBJ/ENA/GenBank under accession numbers CP130552 (DLP4^T^), CP118264 (SDU12^T^), CP130556 (SDU22), CP130555 (GL405^T^), CP130554 (BG257), and CP130553 (MCy1366^T^).

## SUPPLEMENTAL MATERIAL

Supplemental tables and figures spectrum.02535-25-s0001.docx Tables S1 to S7, S9, and S12 and Figs. S1 to S9.

Supplemental tables spectrum.02535-25-s0002.xlsx Tables S8, S10, and S11.

ASM does not own the copyrights to Supplemental Material that may be linked to, or accessed through, an article. The authors have granted ASM a non-exclusive, world-wide license to publish the Supplemental Material files. Please contact the corresponding author directly for reuse.
