# Supplementary material for: Aggregicoccus is a myxobacterial genus inherently deficient in fruiting genes
Source: Microbiol Spectr. 2026 Sep 3;14(10):e02535-25. doi: 10.1128/spectrum.02535-25 (PMC13689315; doi:10.1128/spectrum.02535-25)
Supplement: Supplementary file 1 [file spectrum.02535-25-s0001.docx]

**Supplementary information for**

***Aggregicoccus* is a myxobacterial genus inherently deficient in fruiting genes**

Xinran Du^a^, Jianing Wang^a#^, Jingjing Wang^c^, Yihan Xu^a^, Qi Chen^a^, Zhiwei Su^b#^, Yue-zhong Li^a#^

**Author affiliation**: a. *State Key Laboratory of Microbial Technology, Institute of Microbial Technology,* *Shandong University, Qingdao 266237, P. R. China*

b. *College of Agriculture, Guangxi University, Nanning 530004, P. R. China*

c. *Haide College, Ocean University of China, Qingdao 266100, China*

Xinran Du, 202320446@mail.sdu.edu.cn

**Jianing Wang^#^, wangjianing@sdu.edu.cn**

Jingjing Wang, wangjingjing@ouc.edu.cn

Yihan Xu, Xuyh1025@163.com

Qi Chen, chenqi@sdu.edu.cn

**Zhiwei Su^#^, suzhiwei@gxu.edu.cn; ORCID ID, 0000-0003-2998-8617**

**Yue-zhong Li^#^, lilab@sdu.edu.cn; Tel. (+86) 0532 58631539; ORCID ID, 0000-0001-8336-6638.**

**# The corresponding authors.**

Twelve supplementary tables and nine supplementary figures are available with the online version of this article.

**Supplementary Figures**

**Fig. S1. Phylogenetic trees based on 16S rRNA gene sequences of five novel strains and closely related species. a**, Maximum parsimony tree. The most parsimonious tree (length = 1144) is shown. The consistency index is 0.529720 (0.476654), the retention index is 0.746585 (0.746585), and the composite index is 0.395481 (0.355863), with values for all sites and parsimony-informative sites given in parentheses. **b,** Maximum likelihood tree. The scale bar represents 0.05 substitutions per nucleotide position. Bootstrap values greater than 80%, based on 1,000 replicates, are shown at the nodes. The tree is rooted with *Geobacter daltonii* DSM 22248ᵀ.

**Fig. S2. Gram staining test.**

**Fig. S3. Congo red staining test.** The asterisked side indicates the result after being stained with 0.01% Congo Red aqueous solution. Bars: 1cm.

**Fig. S4. A- and S-motility of *Aggregicoccus* strains. a**, A-motility observed on CY/H plates (1.5% agar) after 24 h. Scale bars: 50 μm. Images captured using a phase-contrast microscope (NIKON ECLIPSE TE2000-S). **b**, S-motility observed on CY/H plates (0.3% agar) after 24 h and 48 h. Scale bars: 1.0 mm. Colonies photographed with a stereomicroscope (SMZ143, Nikon).

**Fig. S5.** **Whole-genome-based phylogenomic tree of cultured myxobacteria species.** The tree was constructed using CVTree3 with protein sequences and K = 6. The red clusters represent the type strains of non-fruiting species that have been reported. The species name *Myxococcus llanfairpwllgwyngyllgogerychwyrndrobwllllantysiliogogogochensis* is abbreviated as *Myxococcus llanfair*.

**Fig. S6. Predatory activity against various bacterial species.** Predation assay conducted on TPM plates over 8 days. Scale bar: 0.5 cm. Target prey species include *Escherichia coli* DH5α, *Pseudomonas aeruginosa* PAO1, *Staphylococcus aureus* DSM 20231, *Micrococcus luteus* ATCC 4698, and *Acinetobacter baumannii* ATCC 19606.

**Fig. S7. Antifungal activity against mycelial growth.** Dual-culture assay conducted on TPY plates for 17 days. Scale bar: 0.5 cm. Target fungi: *Exserohilum turcicum* ACCC 36266, *Septoria lycopersici* ACCC 36106, *Colletotrichum orbiculare* ACCC 36065, and *Curvularia lunata* ACCC 38967.

**Fig. S8. Biocontrol-associated genes in *Aggregicoccus* genomes. a**, Number of genes encoding enzymatic functions. **b**, Phylogenetic tree of the gluM gene and its homologs**. c**, Predation-specific protein families.

**Fig. S9. Global distribution of *Aggregicoccus* in environmental samples.** The number in parentheses indicates the total number of samples. Percentages on the orange bar chart represent the detection frequency of *Aggregicoccus*.

**Supplementary Tables**

**Table S1. Pairwise 16S rRNA gene sequence similarities (%) among *Aggregicoccus* strains.** Values were obtained from the EzTaxon database analysis.

| Blast | DLP4^T^ | SDU12^T^ | SDU22 | GL405^T^ | BG257 | MCy1366^T^ |
| --- | --- | --- | --- | --- | --- | --- |
| DLP4^T^ | 100 |  |  |  |  |  |
| SDU12^T^ | 97.43 | 100 |  |  |  |  |
| SDU22 | 97.97 | 99.41 | 100 |  |  |  |
| GL405^T^ | 96.1 | 97.12 | 96.66 | 100 |  |  |
| BG257 | 96.28 | 97.21 | 96.66 | 99.26 | 100 |  |
| MCy1366^T^ | 97.68 | 98.63 | 98.29 | 97.35 | 97.28 | 100 |

**Table S2. Genome sequencing information and general features of the genomes.**

| Attributes | Accession no. | Size (Mbp) | Reads N50 (bp) | DNA G+C (mol%) | Number of | | | | |
| --- | --- | --- | --- | --- | --- | --- | --- | --- | --- |
|  |  |  |  |  | Reads | Genes | rRNA | sRNA | tRNA |
| DLP4^T^ | CP130552 | 9.15 | 21,125 | 70.73 | 260,730 | 8,065 | 12 | 17 | 74 |
| SDU12^T^ | CP118264 | 8.35 | 14,448 | 70.01 | 314,260 | 7,350 | 12 | 7 | 69 |
| SDU22 | CP130556 | 8.56 | 16,550 | 69.99 | 341,145 | 7,390 | 9 | 7 | 65 |
| GL405^T^ | CP130555 | 8.23 | 10,811 | 70.86 | 823,483 | 6,970 | 9 | 5 | 61 |
| BG257 | CP130554 | 8.39 | 9,723 | 70.94 | 646,933 | 7,128 | 9 | 5 | 61 |
| MCy1366^T^ | CP130553 | 9.69 | 22,534 | 70.46 | 270,851 | 8,381 | 12 | 8 | 73 |

**Table S3. ANI and dDDH values for pairwise comparisons among six *Aggregicoccus* strains.** ANI values (%) are shown above the diagonal, while dDDH values (%) are presented below the diagonal.

| **Strains** | **SDU12^T^** | **SDU22** | **DLP4^T^** | **GL405^T^** | **BG257** | **MCy1366^T^** |
| --- | --- | --- | --- | --- | --- | --- |
| **SDU12^T^** | 100 | 96.6 | 86.42 | 83.45 | 83.37 | 86.26 |
| **SDU22** | 70.5 | 100 | 86.44 | 83.27 | 83.33 | 86.23 |
| **DLP4^T^** | 31 | 31.1 | 100 | 82.95 | 83.1 | 89.53 |
| **GL405^T^** | 26.3 | 26.3 | 25.9 | 100 | 97.08 | 83.05 |
| **BG257** | 26.4 | 26.4 | 25.9 | 74 | 100 | 83.3 |
| **MCy1366^T^** | 30.8 | 30.9 | 37.6 | 26.1 | 26.2 | 100 |

**Table S4. Differential physiological and biochemical characteristics of *Aggregicoccus* strains.** +: positive; –: negative; +w: weakly positive. All strains were positive for esterase (C4), esterase (C8), naphthol-AS-BI-phosphohydrolase, leucine arylamidase, valine arylamidase, acid phosphatase. All strains were negative for hydrolysis of chitin and cellulose, cystine arylamidase, trypsin, α-galactosidase, β-galactosidase, β-glucuronidase, α-glucosidase, β-glucosidase, N-acetyl-β-glucosaminidase, α-mannosidase, α-fucosidase; citrate, hydrogen sulfide, urease, indole production, acetyl methylcarbinol, glucose, mannose, rhamnose, sucrose, melibiose, arginine dihydrolase, p-Nitrophenyl-β-D-galactopyranosidase, assimilation of: glucose, arabinose, mannose, mannitol, N-acetyl-D-glucosamine, maltose, potassium gluconate, capric acid, adipic acid, malic acid, sodium citrate, phenylacetic acid.

| **Characteristics** | **DLP4^T^** | **SDU12^T^** | **SDU22** | **GL405^T^** | **BG257** | **MCy1366^T^** |
| --- | --- | --- | --- | --- | --- | --- |
| **Growth temperature range (℃)** | 18−45 | 18−40 | 18−40 | 18−35 | 18−35 | 18−35 |
| **Growth pH range** | 6.0−10.0 | 6.5−10.0 | 6.5−10.0 | 6.0−10.0 | 6.0−10.0 | 6.0−9.0 |
| **NaCl range for growth (%, w/v)** | 0−1.5 | 0−1.5 | 0−0.5 | 0−1.5 | 0−1.0 | 0−0.5 |
| **Gram stain** | − | − | − | − | − | − |
| **Congo red reaction** | + | + | + | +w | +w | + |
| **Hydrolysis of** |  |  |  |  |  |  |
| casein, starch, gelatin | + | + | + | + | + | + |
| chitin, cellulose | − | − | − | − | − | − |
| **Enzymes activity** |  |  |  |  |  |  |
| Oxidase | + | − | − | +w | +w | − |
| Catalase | + | + | + | ++ | ++ | ++ |
| Urease | − | − | − | − | − | − |
| Tryptophan | − | + | + | + | + | − |
| Lipase (C14) | + | + | + | + | + | − |
| α-chymotrypsin | + | + | + | − | − | + |
| Nitrate reduction | + | + | + | − | − | − |
| Aesculin hydrolysis | + | − | − | + | + | + |

**Table S5. Cellular fatty acid content of *Aggregicoccus*.** Data are expressed as percentages of total fatty acids. Major fatty acids ( > 5 %) are indicated in bold; –, not detected. *Summed features are groups of two or three fatty acids that are not separated by GLC with the Sherlock Microbial Identification System. Summed feature 3 contained C_16 : 1_ω7c /C_16 : 1_ω6c. Summed Feature 4 contained iso-C_17 : 1_ I /anteiso B C_17 : 1._

| **Fatty acids** | **DLP4^T^** | **SDU12^T^** | **SDU22** | **GL405^T^** | **BG257** | **MCy1366^T^** |
| --- | --- | --- | --- | --- | --- | --- |
| Straight chain |  |  |  |  |  |  |
| C_12 : 0_ | 0.2 | 0.1 | **–** | 0.2 | 0.2 | 0.2 |
| C_14 : 0_ | 0.2 | 1.3 | 0.4 | 0.5 | 0.6 | 1.1 |
| C_16 : 0_ | 1.2 | **15.2** | 2.6 | **13.6** | **16.1** | **20.5** |
| C_18 : 0_ | 0.3 | 4.52 | 0.2 | **5.3** | **7.2** | **6.9** |
| Branched chain |  |  |  |  |  |  |
| iso-C_13 : 0_ | 0.4 | 0.3 | 0.6 | 0.3 | 0.2 | 0.2 |
| iso-C_14 : 0_ | 0.4 | **–** | 0.2 | 0.3 | 0.1 | **–** |
| iso-C_15 : 0_ | **35.8** | **16.9** | **27.4** | **15.8** | **17.8** | **14** |
| iso-C_16 : 0_ | **10.5** | 0.4 | 2.2 | 3.9 | 2 | 1.1 |
| iso-C_17 : 0_ | **6.8** | **14.1** | **20.6** | **9.7** | **10.1** | 4 |
| anteiso C_16 : 0_ | 0.7 | 0.1 | 0.3 | 0.1 | 0.2 | **–** |
| iso-C_17: 1_ω10c | 3.2 | 0.2 | 1.7 | 1.6 | 0.4 | 0.2 |
| C_18: 1_ω9c | 0.5 | **17.5** | 0.3 | **16** | **11.2** | **19.1** |
| C_16: 1_ω9c | **–** | 1.9 | 0.2 | – | – | **–** |
| C_16: 1_ω5c | **7.4** | 4.5 | **7.3** | 2.2 | 3.9 | 0.8 |
| iso-C_17 : 0_ 3-OH | 2.7 | 1 | 0.7 | 0.8 | 1.1 | 0.9 |
| *Summed Feature 3 | 2.7 | **18.8** | **8.8** | **23.2** | **19.5** | **27.6** |
| *Summed Feature 4 | **20.4** | 0.9 | **10.9** | 1.6 | 1.7 | 0.2 |
| *Summed Feature 8 | **–** | **–** | **8.1** | 0.9 | 0.8 | 1 |

**Table S6. Antibiotic resistance profiling of *Aggregicoccus*.** +: positive for resistance; −: negative for resistance; +w: weakly positive.

| **Characteristics** | **DLP4^T^** | **SDU12^T^** | **SDU22** | **GL405^T^** | **BG257** | **MCy1366^T^** |
| --- | --- | --- | --- | --- | --- | --- |
| **Antibiotic tolerance (50 μg/ml )** |  |  |  |  |  |  |
| Tobramycin | − | +w | − | − | − | + |
| Vancomycin hydrochloride | − | − | − | − | − | − |
| Ampicillin | − | − | − | − | − | − |
| Carbenicillin | + | + | + | + | + | + |
| Rifampin | + | + | + | + | + | + |
| Kanamycin | − | + | + | − | − | + |
| Cefalothin Sodium | − | − | − | − | − | − |
| Oxytetracycline | _ | − | − | − | − | − |
| Gentamicin | − | + | + | − | − | + |
| Spectinomycin | − | − | + | − | − | − |
| Chloramphenicol (34 μg/ml) | − | − | − | − | − | − |
| Daptomycin | + | + | + | + | + | + |

**Table S7. Genome information of the type strains of *Myxococcus*.** The species name *Myxococcus llanfairpwllgwyngyllgogerychwyrndrobwllllantysiliogogogochensis* is abbreviated as *Myxococcus llanfair*.

| **Attributes** | **Accession no.** | **Size (Mbp)** | **Contigs N50** | **DNA G+C (mol%)** | **Number of** | |
| --- | --- | --- | --- | --- | --- | --- |
|  |  |  |  |  | **Contigs** | **Genes** |
| *Myxococcus eversor* (AB053B^T^) | GCA_010894455.1 | 11.39 | 313.1 kb | 69 | 135 | 9,105 |
| *Myxococcus qinghaiensis* (QH3KD-4-1^T^) | GCA_024198215.1 | 11.52 | 323.4 kb | 69 | 88 | 9,203 |
| *Myxococcus llanfair.* (AM401^T^) | GCA_006636215.1 | 12.41 | 35.7 kb | 68.5 | 1077 | 10,403 |
| *Myxococcus fulvus* (DSM 16525^T^) | GCA_900111765.1 | 10.82 | 755.5 kb | 70 | 43 | 8,658 |
| *Myxococcus guangdongensis* (K38C18041901^T^) | GCA_024198255.1 | 11.2 | 554.7 kb | 70 | 53 | 8,942 |
| *Myxococcus stipitatus* (DSM 14675^T^) | GCA_000331735.1 | 10.35 | 10.4 Mb | 69 | 1 | 8,128 |
| *Myxococcus dinghuensis* (K15C1803190^T^) | GCA_024198235.1 | 10.41 | 207.4 kb | 71 | 124 | 8,560 |
| *Myxococcus vastator* (AM301^T^) | GCA_010894475.1 | 8.99 | 15.9 kb | 70 | 1008 | 7,753 |
| *Myxococcus virescens* (DSM 2260^T^) | GCA_900101905.1 | 9.23 | 325.1 kb | 69 | 68 | 7,602 |
| *Myxococcus xanthus* (DSM 16526^T^) | GCA_900106535.1 | 9.26 | 9 Mb | 69 | 1 | 7,142 |

**Table S8. Blastp results for identification of homologues for proteins involved in the *Myxococcus xanthus* social lifestyle in *Aggregicoccus* and *Myxococcus* genomes.** “#” indicates that the genome is a draft genome. The species name *Myxococcus llanfairpwllgwyngyllgogerychwyrndrobwllllantysiliogogogochensis* is abbreviated as *Myxococcus llanfair.*

The content of the table can be found in the Excel file.

**Table S9. The genomes of cultured myxobacterial species that were reported to be unable to fruit (excluding *Aggregicoccus*).**

| **Strian ID** | **Accession no.** | **Genome size (bp)** | **GC (%)** | **Taxonomy** | **Reference** |
| --- | --- | --- | --- | --- | --- |
| *Simulacricoccus ruber* (17bor-14^T^) | GCA_009659535.1 | 6,926,196 | 73 | Bacteria;Myxococcota;Myxococcia;Myxococcales;Myxococcaceae;Simulacricoccus | 10.1099/ijsem.0.002936 |
| *Vulgatibacter incomptus* (DSM 27710^T^) | GCA_001263175.1 | 4,350,553 | 69 | Bacteria;Myxococcota;Myxococcia;Myxococcales;Vulgatibacteraceae;Vulgatibacter | 10.1099/ijs.0.063198-0 |
| *Anaeromyxobacter paludicola* (Red630^T^) | GCA_023169965.1 | 3,262,476 | 68.2 | Bacteria;Myxococcota;Myxococcia;Myxococcales;Anaeromyxobacteraceae | 10.1099/ijsem.0.005546 |
| *Anaeromyxobacter diazotrophicus* (Red267^T^) | GCA_013340205.1 | 4,829,832 | 74.49 | Bacteria;Myxococcota;Myxococcia;Myxococcales;Anaeromyxobacteraceae | 10.1099/ijsem.0.005546 |
| *Anaeromyxobacter soli* (SG29^T^ ) | GCA_022808855.1 | 5,308,032 | 73.5 | Bacteria;Myxococcota;Myxococcia;Myxococcales;Anaeromyxobacteraceae | 10.1099/ijsem.0.006268 |
| *Anaeromyxobacter terrae* (SG22^T^) | GCA_022690685.1 | 5,655,738 | 73 | Bacteria;Myxococcota;Myxococcia;Myxococcales;Anaeromyxobacteraceae | 10.1099/ijsem.0.006268 |
| *Anaeromyxobacter oryzisoli* (SG63^T^) | GCA_022690765.1 | 5,313,002 | 73.5 | Bacteria;Myxococcota;Myxococcia;Myxococcales;Anaeromyxobacteraceae | 10.1099/ijsem.0.006268 |
| *Anaeromyxobacter oryzae* (Red232^T^) | GCA_023169945.1 | 6,720,545 | 73.5 | Bacteria;Myxococcota;Myxococcia;Myxococcales;Anaeromyxobacteraceae | 10.1099/ijsem.0.005546 |
| *Anaeromyxobacter dehalogenans* (2CP-1^T^) | GCA_000022145.1 | 5,029,329 | 74.5 | Bacteria;Myxococcota;Myxococcia;Myxococcales;Anaeromyxobacteraceae | 10.1128/AEM.68.2.893–900.2002 |
| *Polyangium aurulentum* (SDU3-1^T^) | GCA_005144635.2 | 12,320,079 | 69.5 | Bacteria;Myxococcota;Polyangia;Polyangiales;Polyangiaceae;Polyangium | 10.1016/j.syapm.2021.126274 |
| *Byssovorax cruenta* (JCM 12614^T^) | GCA_001312805.1 | 5,987,311 | 66.5 | Bacteria;Myxococcota;Polyangia;Polyangiales;Polyangiaceae;Byssovorax | 10.1099/ijs.0.63628-0 |
| *Labilithrix luteola* (DSM 27648^T^) | GCA_001263205.1 | 12,191,466 | 66 | Bacteria;Myxococcota;Polyangia;Polyangiales;Polyangiaceae;Labilithrix | 10.1099/ijs.0.063198-0 |
| *Sandaracinus amylolyticus* (DSM 53668^T^) | GCA_000737325.2 | 10,327,335 | 72 | Bacteria;Myxococcota;Polyangia;Polyangiales;Sandaracinaceae;Sandaracinus | 10.1099/ijs.0.033696-0 |

**Table S10. Blastp results for identification of homologues for proteins involved in the *Myxococcus xanthus* social lifestyle in in the cultured non-fruiting myxobacteria (excluding *Aggregicoccus*).** “#” indicates that the genome is a draft genome.

The content of the table can be found in the Excel file.

**Table S11. Blastp results for identification of killing systems for predation.**

The content of the table can be found in the Excel file.

**Table S12. The biosynthetic gene clusters of *Aggregicoccus* spp. predicted by antiSMASH.**

| **Type** | **SDU12^T^** | **SDU22** | **DLP4^T^** | **GL405^T^** | **BG257** | **MCy1366 ^T^** |
| --- | --- | --- | --- | --- | --- | --- |
| Terpene | 4 | 4 | 3 | 4 | 4 | 4 |
| RRE-containing | 4 | 5 | 3 | 3 | 3 | 4 |
| Lanthipeptide-class-i, ii | 5 | 4 | 2 | 4 | 4 | 1 |
| NRPS/NRPS-like | 5 | 5 | 3 | 5 | 5 | 6 |
| thioamitides | 1 | 1 | 1 | 2 | 2 | 1 |
| LAP | 1 | 1 | 1 | 1 | 1 | 1 |
| T1PKS | 1 | 1 | 0 | 3 | 3 | 1 |
| RiPP-like | 1 | 1 | 2 | 1 | 2 | 1 |
| T3PKS | 1 | 1 | 1 | 2 | 2 | 1 |
| microviridin | 1 | 1 | 1 | 1 | 1 | 1 |
| NRPS-independent-siderophore | 1 | 1 | 1 | 0 | 0 | 0 |
| arylpolyene | 1 | 2 | 0 | 0 | 0 | 0 |
| resorcinol | 1 | 1 | 0 | 0 | 0 | 0 |
| betalactone | 1 | 1 | 0 | 0 | 0 | 0 |
| butyrolactone | 1 | 0 | 0 | 0 | 0 | 0 |
| linaridin | 0 | 0 | 1 | 0 | 0 | 0 |
| acyl_amino_acids | 0 | 0 | 0 | 1 | 1 | 0 |
| thiopeptide | 0 | 0 | 0 | 1 | 1 | 0 |
| NRP-metallophore | 0 | 0 | 0 | 1 | 1 | 1 |
| Indole | 1 | 1 | 0 | 1 | 1 | 0 |
| Total | 30 | 30 | 19 | 30 | 31 | 22 |
